# Implications of the COVID-19 pandemic on self-reported health status and noise annoyance in rural and non-rural Canada

**DOI:** 10.1038/s41598-022-19907-w

**Published:** 2022-09-24

**Authors:** David S. Michaud, Leonora Marro, Allison Denning, Shelley Shackleton, Nicolas Toutant, Emily Cameron-Blake, James P. McNamee

**Affiliations:** 1 Health Canada, Environmental and Radiation Health Sciences Directorate, Consumer and Clinical Radiation Protection Bureau, Non-Ionizing Radiation Health Sciences Division, 775 Brookfield Road, Ottawa, ON K1A1C1 Canada; 2 Biostatistics Section, Health Canada, Environmental and Radiation Health Sciences Directorate, Environmental Health Science and Research Bureau, 251 Sir Frederick Banting Driveway, Tunney’s Pasture, Ottawa, ON K1A0K9 Canada; 3 Public Engagement, Research and Analysis Division, Health Canada, Communications and Public Affairs Branch, 200 Eglantine Driveway, Tunney’s Pasture, Ottawa, ON K1A 0K9 Canada; 4 Advanis, Inc., 3981 Boulevard Saint-Laurent, Suite 200, Montréal, QC H2W 1Y5 Canada; 5 Blavatnik School of Government, University of Oxford, Radcliffe Observatory Quarter, Woodstock Road, Oxford, OX2 6GG UK

**Keywords:** Environmental social sciences, Psychology and behaviour

## Abstract

The *Canadian Perspectives on Environmental Noise Survey* (CPENS), conducted between April 12th, 2021 and May 25th, 2021 coincided with the third wave of the COVID-19 pandemic. Canadians 18 years of age and older (n = 6647) reported the degree to which the pandemic affected their physical health, mental health, stress, annoyance toward environmental and indoor noise, and overall well-being. Depending on the outcome evaluated, between 18 and 67% of respondents reported the measure as “somewhat” or “much worse” due to the pandemic. Stress was most affected, followed by mental health, overall well-being, physical health, annoyance toward environmental noise and annoyance toward indoor noise. Logistic regression models indicated that province, geographic region (rural/remote, suburban, urban), age, gender, poor physical/mental health, heart disease, a history of high sleep disturbance (in general) or diagnosed sleep disorders, anxiety/depression, working/schooling from home, and being retired significantly impacted the odds of reporting a worsening by the pandemic to varying degrees and directions, depending on the outcome. Indigenous status was unrelated to any of the modelled outcomes. Future research could address some of the noted study limitations and provide the data to determine if the observations on the reported measures of health are temporary, or long-lasting.

## Introduction

The *Canadian Perspectives on Environmental Noise Survey* (CPENS) conducted between April 12th, 2021 and May 25th 2021, investigated attitudes, expectations and perceptions of environmental noise in rural and non-rural Canada^1^. Because this period coincided with the 3^rd^ wave of the severe acute respiratory syndrome coronavirus 2 (SARS-CoV-2, hereinafter COVID-19) global pandemic, the potential influence that the pandemic may have had on survey responses was considered. During the survey data collection period, the pandemic experience across Canada varied from province to province. The average daily number of new cases, admissions to intensive care units, and death in Canada during this period was 6880, 1249, and 45.3, respectively^2^. Global efforts to manage the spread of COVID-19 included government-mandated restrictions on public gatherings, festivals, concerts, and travel by air, rail and automobiles. Industrial activities, including construction, were curtailed in some circumstances. These unprecedented restrictions on human activities had impacts on the environment that included a reduction in environmental noise levels^3–6^. Changes in environmental and/or indoor noise levels (e.g. due to greater occupancy during stay-at-home orders) also influenced reported annoyance^7, 8^.

Noise annoyance can be broadly viewed as a self-reported adverse response to unwanted noise, which subsumes a variety of reactions that bother, disturb or annoy an individual^9^. Protracted high magnitudes of noise annoyance is considered a health effect of environmental noise by the World Health Organization^10, 11^ and a serious contributor to the environmental burden of disease^12^. Dümen and Şaher^8^ reported significant decreases in annoyance toward road traffic noise during lockdown periods and opposite patterns toward noise originating from other rooms within one’s own home. As expected, the authors found that self-reported stress (in general over the previous month) as measured on the abbreviated version of the Perceived Stress Scale^13^ and anxiety were elevated during the pandemic. Both measures were positively correlated with noise annoyance, which has been reported elsewhere^12, 14–16^.

Beyond changes to the physical acoustical environment and the potential for this to mitigate/exacerbate annoyance^7, 8^, the global pandemic has been associated with effects on mental health. Samji et al.^17^ data aligned with Statistics Canada^18^ where COVID-19-related adverse impacts on self-reported mental health were more pronounced among younger adults. A deterioration in mental health and stress associated with the pandemic was particularly noted among younger individuals in the study by Cost et al.^19^. However, in the Statistics Canada survey, the prevalence of rating physical health as “very good” or “excellent” *increased* by nearly 10% during the pandemic compared to pre-pandemic levels^18^. In their review of the research on changes in mental health associated with the pandemic, Vindegaard and Benros^20^ identified studies that reported living in rural versus urban areas as a risk factor for increased anxiety and/or depression.

The objectives of the current CPENS analysis were to explore the variables related to self-reported impairments in physical health, mental health, stress, environmental noise annoyance, indoor noise annoyance and overall well-being. It was of particular interest to investigate the relationship between these measures and geographic region (i.e. rural/remote, suburban and urban). CPENS also aimed to evaluate the impact of the pandemic on Indigenous Canadians. Indigenous Peoples are kinship-oriented, making the public health measures implemented to curb the transmission of COVID-19 particularly challenging^21^. It was hypothesized that Indigenous respondents may report greater impacts of the pandemic on self-reported measures of health.

## Methods

### Sample design

#### Sample recruitment and response rate

A detailed presentation of CPENS methodology is provided by Michaud et al.^1^. Briefly, a general population probability-based random sample (GPRS) from all provinces was used to recruit respondents via telephone to the online survey. For this study, the sample was created using two approaches. A random digit dialing approach (i.e., GPRS) for the general population across the country where the sample was pulled randomly by province proportionally to their size nationally, and by postal codes of the First Nations and remote areas in order to oversample those specific groups. Non-respondents that did not complete the survey were sent a reminder message at 3 and 6 days after the initial recruitment. Of the 22,892 potentially eligible participants, 11,492 were recruited to the survey, for a recruitment rate of 50.6%. Of the 11,492 recruited participants, 6647 completed the online survey, for an overall response rate among eligible respondents of 29.0%. To achieve a representative sample of rural, urban, and suburban areas, survey data were weighted with the most recent Statistics Canada census data. This also corrected for over and under sampled groups in certain geographic locations. There was no evidence of extreme values in the weighted data that would indicate a sampling bias. The margin of error for the study was ± 1.2%, at a 95% confidence level (i.e., 19 times out of 20).

#### Determining geographic sampling regions

The sampling frame was set to target respondents from remote/rural, suburban and urban areas in all ten Canadian provinces using the forward sortation area (FSA) postal code information^22^. Respondents indicated the geographic region that best corresponded to the area in which they lived based on population size. Because some postal codes can be both rural and urban, geographic region in the statistical analysis was based on self-reported geographic region.

#### Questionnaire development, pretesting and quality control

The questionnaire included content to evaluate noise perception, annoyance, and expectations of quiet, health-related and socio-demographic variables. The average length of time to complete the online questionnaire was just under 10 min. The questionnaire was designed by Health Canada and pre-tested in both English and French. For the pretesting, 299 people were recruited by phone (212 in English and 87 in French). This led to 72 completed online surveys (61 English, and 11 French). Minor changes made to the survey after pre-testing did not affect the pre-test data, allowing results collected during the pre-test to be included in the final analysis. The English and French versions of the survey are available through Library and Archives Canada^23^.

### Definitions

In CPENS, participants were asked to indicate how they have been personally affected by the COVID-19 pandemic with respect to physical health, mental health, annoyance toward environmental noise, annoyance toward indoor noise, stress in their life, and overall well-being. Response categories for these six outcome variables were as follows: much worse, somewhat worse, unchanged, somewhat improved, and much improved. For modelling, the responses were grouped as: “somewhat/much worse” and “unchanged/somewhat/much improved”. When reporting prevalence rates the responses were grouped into the three following categories: “somewhat/much worse”, “unchanged” and “somewhat/much improved”. A number of other variables were collected in CPENS that were considered to be potentially associated to the six evaluated outcomes. These included the demographic variables such as age, gender, education, income and Indigenous status. Age in years was divided into three groups (18–34, 35–54, 55 +). The following gender categories were defined (female, male, other/prefer not to say). Education was rated as: up to high school diploma or equivalent, certificate or diploma, bachelor's degree or post graduate degree. A certificate or diploma could be from a registered apprenticeship, or other trade, college, CEGEP (i.e., Quebec College) or other non-university, university below bachelor's level. Total household income in Canadian dollars was grouped as follows: under $40 K, $40 K to just under $80 K, $80 K to just under $150 K, $150 K and above. Indigenous status was grouped as follows: Self identify as First Nation/ Métis/Inuk (Inuit), or Do not self identify. Province of residence as well as geographic region were also considered as potential predictor variables since the response to the pandemic differed by province as well as geographic region. Due to the smaller sample sizes, the Prairie Provinces (i.e., Manitoba and Saskatchewan), were grouped together as were the Atlantic Provinces (i.e., New Brunswick, Nova Scotia, Prince Edward Island and Newfoundland & Labrador). The remaining provinces (British Columbia, Alberta, Ontario and Quebec) were classified independently. Self-reported geographic region was defined as rural/remote (i.e., < 1000 to 10,000 inhabitants), suburban (i.e., a mixed-use or residential area, existing either as part of a city or urban area, or as a separate residential community within commuting distance of a city) and urban (i.e., 10,000 + inhabitants).

A respondent’s current work or school situation was also considered. Respondents self-identified as follows: working or attending school outside their home; working or attending school inside their home; retired; unemployed; and a portion of those indicating “other” could be grouped as on paid leave (i.e., sick, maternity, and disability). More than one option could be selected; therefore, each situation was considered separately as a “Yes/No” response.

Other variables considered included, sleep disturbance (for any reason at home over the previous 12 months), classified as highly sleep disturbed (rating 8 to 10) versus not highly sleep disturbed (rating 0 to 7). Similarly, sensitivity to noise was defined as highly sensitive to noise (rating 8 to 10) versus not highly sensitive to noise (rating 0 to 7). Participants were asked to rate their overall physical health relative to someone of their age, and their overall mental health (no reference to age). For both of these questions the responses included the following: poor; fair; good; very good; and excellent. These were collapsed as: poor/fair and good/very good/excellent. Heart disease including high blood pressure, anxiety or depression, sleep disorder, and hearing loss were also evaluated as diagnosed by a healthcare professional, not diagnosed but suffer from the condition, or does not apply. Affirming a diagnosis was assumed to indicate the condition was current, and not one that historically existed, but no longer current.

### Statistical methodology

Weighted frequencies and cross-tabulations were used to explore the distribution of demographics and characteristics of the population by Indigenous status and geographic region. Cross-tabulations of each of the health-related outcomes and noise annoyance variables affected by the pandemic with Indigenous status and geographic region were also considered. Chi-square tests of independence compared Indigenous status to non-Indigenous respondents, as well as geographic regions.

Initial univariate logistic regression models were used to investigate the relationship between each of the health-related outcomes, including noise annoyance variables and other variables of interest, as mentioned above. Unadjusted odds ratios (ORs) are reported for each relationship in Supplemental Material (see Table S1). Finally, a multivariate logistic regression model was developed using stepwise regression techniques with a significance level of the chi-square for entering an effect into the model equal to 20% and the significance level of the chi-square for an effect to remain in the model of 5%. Adjusted ORs are reported for the final models for each evaluated outcome affected by the pandemic. Confidence intervals (CI) of ORs including the value 1 indicate insufficient evidence to observe an association between the outcome evaluated and variable under investigation.

Statistical analysis was performed using SAS Enterprise Guide 7.15 (SAS Institute Inc., Cary, NC). A 0.05 statistical significance level was implemented throughout unless otherwise stated. In addition, Bonferroni corrections were made to account for all pairwise comparisons to ensure that the overall Type I (false positive) error rate was less than 0.05. Estimates with a coefficient of variation (CV) between 16.6 and 33.3% were designated “E” and must be interpreted with caution due to the high sampling variability associated with it; CV estimates that exceeded 33.3% were designated “F” indicating that these data could not be released due to questionable validity. No results are reported for cell frequencies less than 10.

This study was approved by the Health Canada and Public Health Agency of Canada Review Ethics Board (Protocol no. REB 2020-038H). Informed consent is implied in the voluntary response to the survey questionnaire. This research was conducted in accordance with all relevant Government of Canada guidelines and regulations for conducting online surveys.

## Results

### Description of survey sample

Population prevalence rates of outcomes from the survey questionnaire and their distribution by Indigenous status and geographic region are summarized in Table 1. A plurality of the participants were 55 years of age or older (38.6%), were female (50.6%), had a bachelor/post graduate degree (44%), had household incomes of $80 K to < $150 K per year (35.9%) and were from Ontario (40.3%). Overall, 16.4% of the study population rated their physical health in general as “fair/poor”. Indigenous respondents were more likely than non-Indigenous respondents to rate their physical health as fair/poor (i.e., 24.1% vs 16%, respectively, p < 0.05). Similarly, 21.1% of the study population rated their mental health as fair/poor with an increased proportion among Indigenous peoples (29.7%) rating their mental health as fair/poor (compared to non-Indigenous at 20.6%, p < 0.05). High sleep disturbance was reported by 7.8% of the population overall, 9% among Indigenous respondents and 9.8% among urban dwellers. High noise sensitivity was reported by 13.4% of the population with similar levels across both Indigenous and non-Indigenous groups and geographic regions.

**Table 1:** Weighted prevalence (%) and (95% confidence interval) of population characteristics by Indigenous status and geographic region.

	Frequency (n)	Overall	Indigenous^f^	Non-Indigenous	Rural/remote	Suburban	Urban
**Age (years)**							
18 to 34	1814	27.3 (26.2–28.4)	22.6 (18.3–27.5)	27.5 (26.4–28.6)	23.8 (21.4–26.4)	27.5 (26.1–29.1)	28.7 (26.8–30.6)
35 to 54	2267	34.1 (33–35.3)	43.7 (38.3–49.2)	33.6 (32.5–34.8)^g^	36.3 (33.6–39.2)	33.9 (32.3–35.5)	33.3 (31.3–35.3)
55 or older	2566	38.6 (37.4–39.8)	33.7 (28.7–39.1)	38.8 (37.7–40.1)	39.8 (37–42.7)	38.6 (36.9–40.2)	38 (36–40.1)
**Gender**							
Female	3366	50.6 (49.4–51.8)	55.2 (49.7–60.5)	50.4 (49.2–51.6)	50.8 (47.9–53.7)	52 (50.3–53.7)	48.5 (46.4–50.6)
Male	3193	48 (46.8–49.2)	42.8 (37.5–48.3)	48.3 (47.1–49.5)	48.1 (45.2–51)	46.9 (45.2–48.6)	49.8 (47.7–51.9)
Other/prefer not to say	88	1.3 (1.1–1.6)	F	1.3 (1–1.6)	1.1 (0.7–1.9) E	1.1 (0.8–1.5)	1.8 (1.3–2.4)
**Current situation regarding work or schooling**							
Work/attend school outside home	2413	36.3 (35.2–37.5)	40.6 (35.3–46.1)	36.1 (34.9–37.3)	43.3 (40.4–46.2)	34.9 (33.3–36.6)^h^	34.8 (32.8–36.8)^h^
Work/attend school inside home	2331	35.1 (33.9–36.2)	29.5 (24.7–34.7)	35.4 (34.2–36.5)^g^	24.7 (22.3–27.3)	37.8 (36.1–39.4)^h^	36.4 (34.4–38.4)^h^
Retired	1501	22.6 (21.6–23.6)	15.8 (12.2–20.2)	22.9 (21.9–24)^g^	23.5 (21.1–26)	22.8 (21.4–24.3)	21.8 (20.1–23.5)
Unemployed	538	8.1 (7.5–8.8)	12.4 (9.2–16.5)	7.9 (7.2–8.6)^g^	9.5 (7.9–11.4)	7.6 (6.8–8.6)	8 (7–9.3)
On paid leave (sick leave, maternity, disability)	180	2.7 (2.3–3.1)	4.9 (3–7.9) E	2.6 (2.2–3)^g^	2.9 (2–4) E	2.8 (2.3–3.4)	2.5 (1.9–3.2)
Other	221	3.3 (2.9–3.8)	3.9 (2.3–6.7) E	3.3 (2.9–3.8)	4.5 (3.4–5.9)	3 (2.5–3.7)	3.2 (2.5–4)
**Highest level of education completed**							
High school/equiv.^a^	1221	18.7 (17.8–19.7)	24.8 (20.3–30)	18.4 (17.5–19.4)^g^	23.5 (21.1–26.1)	18.4 (17.1–19.8)^h^	16.7 (15.2–18.4)^h^
Cert/dip < bachelor^b^	2429	37.3 (36.1–38.4)	47.5 (41.9–53)	36.7 (35.6–38)^g^	45.5 (42.5–48.4)	35.8 (34.2–37.5)^h^	35.2 (33.2–37.2)^h^
Bachelor/post graduate degree	2870	44 (42.8–45.2)	27.7 (23–33)	44.8 (43.6–46.1)^g^	31 (28.4–33.8)	45.8 (44.1–47.5)^h^	48.1 (46–50.2)^h^
Prefer not to say	147						
**Total household income in 2020 from all sources before taxes**							
< $40 K	1069	18.4 (17.4–19.4)	27.5 (22.7–32.9)	17.9 (16.9–18.9)^g^	20.4 (18–23)	16.3 (15–17.7)^h^	20.3 (18.6–22.2)
$40 K–< $80 K	1629	28 (26.8–29.1)	29.2 (24.2–34.7)	27.9 (26.8–29.1)	30.5 (27.7–33.4)	26.4 (24.8–28.1)	28.9 (27–31)
$80 K–< $150 K	2090	35.9 (34.7–37.1)	30 (25–35.5)	36.2 (35–37.5)^g^	33.9 (31.1–36.9)	38.5 (36.7–40.3)^h^	33.1 (31–35.2)
> $150 K	1034	17.8 (16.8–18.8)	13.3 (9.9–17.7)	18 (17–19)^g^	15.2 (13.1–17.5)	18.8 (17.4–20.2)^h^	17.6 (16–19.4)
Prefer not to say	756						
**Province**							
BC	955	14.4 (13.6–15.3)	21.9 (17.7–26.8)	14 (13.2–14.9)^g^	14.6 (12.7–16.8)	15.6 (14.4–16.9)	12.5 (11.2–14)
AB	803	12.1 (11.4–12.9)	21.3 (17.1–26.1)	11.7 (10.9–12.5)^g^	12.1 (10.3–14.1)	10.4 (9.4–11.4)	14.8 (13.4–16.3)
MB/SK	329	5 (4.5–5.5)	14.2 (10.8–18.5)	4.5 (4–5)^g^	5.7 (4.5–7.2)	3.3 (2.8–4)	7.1 (6.1–8.2)
ON	2670	40.3 (39.1–41.5)	22.8 (18.5–27.8)	41.2 (40–42.4)^g^	33.3 (30.7–36.1)	41.6 (39.9–43.3)^h^	42 (40–44.1)^h^
QC	1234	18.6 (17.7–19.6)	11.8 (8.7–15.8)	19 (18–20)^g^	20.3 (18–22.7)	19.7 (18.3–21)	16.2 (14.7–17.8)^h^
Atlantic provinces^c^	635	9.6 (8.9–10.3)	8 (5.5–11.5) E	9.7 (9–10.4)	14 (12.1–16.1)	9.5 (8.6–10.6)^h^	7.4 (6.4–8.6)^h^
Prefer not to say	18						
**Rated physical health in general, for one’s age**							
Fair/poor	1091	16.4 (15.5–17.3)	24.1 (19.7–29.1)	16 (15.1–17)^g^	16.8 (14.7–19.1)	16.5 (15.3–17.8)	16 (14.5–17.6)
Excellent/very good/good	5556	83.6 (82.7–84.5)	75.9 (70.9–80.3)	84 (83–84.9)^g^	83.2 (80.9–85.3)	83.5 (82.2–84.7)	84 (82.4–85.5)
**Rated mental health in general, for one’s age**							
Fair/poor	1399	21.1 (20.1–22)	29.7 (24.9–34.9)	20.6 (19.6–21.6)^g^	20.8 (18.5–23.2)	20.9 (19.5–22.3)	21.5 (19.8–23.2)
Excellent/very good/good	5248	78.9 (78–79.9)	70.3 (65.1–75.1)	79.4 (78.4–80.4)^g^	79.2 (76.8–81.5)	79.1 (77.7–80.5)	78.5 (76.8–80.2)
HSD^d^	516	7.8 (7.1–8.4)	9 (6.3–12.6)E	7.7 (7.1–8.4)	5.5 (4.3–6.9)	7.2 (6.4–8.1)	9.8 (8.6–11.1)^h^
Highly sensitive to noise^e^	893	13.4 (12.6–14.3)	13.5 (10.2–17.7)	13.4 (12.6–14.3)	12 (10.3–14.1)	13.9 (12.7–15.1)	13.5 (12.1–15)
**Chronic health conditions diagnosed by professional or undiagnosed but suffering**							
Heart disease incl. high blood pressure							
Diagnosed	1265	19 (18.1–20)	19.1 (15.1–23.8)	19 (18.1–20)	19.4 (17.2–21.8)	19.7 (18.4–21.1)	17.8 (16.3–19.5)
Suffer	214	3.2 (2.8–3.7)	4.7 (2.9–7.7)E	3.1 (2.7–3.6)	4.1 (3.1–5.4)	3.1 (2.5–3.7)	3 (2.3–3.8)
Not applicable	5168	77.7 (76.7–78.7)	76.2 (71.2–80.6)	77.8 (76.8–78.8)	76.5 (74–78.9)	77.2 (75.8–78.6)	79.2 (77.4–80.8)
Anxiety or depression							
Diagnosed	1357	20.4 (19.5–21.4)	30.2 (25.4–35.5)	19.9 (19–20.9)^g^	19.8 (17.5–22.2)	19.9 (18.6–21.3)	21.5 (19.8–23.3)
Suffer	1355	20.4 (19.4–21.4)	23.3 (19–28.3)	20.2 (19.3–21.2)	19.9 (17.7–22.3)	21.1 (19.8–22.5)	19.5 (17.9–21.2)
Not applicable	3935	59.2 (58–60.4)	46.4 (41–51.9)	59.8 (58.6–61)^g^	60.3 (57.5–63.1)	59 (57.3–60.6)	59 (56.9–61)
Sleep disorder							
Diagnosed	766	11.5 (10.8–12.3)	14.6 (11.2–18.9)	11.4 (10.6–12.2)	10.5 (8.9–12.5)	12.4 (11.3–13.6)	10.7 (9.5–12.1)
Suffer	1171	17.6 (16.7–18.6)	21.9 (17.7–26.8)	17.4 (16.5–18.4)	17.9 (15.8–20.2)	17 (15.7–18.3)	18.5 (16.9–20.2)
Not applicable	4710	70.9 (69.7–71.9)	63.4 (58–68.6)	71.2 (70.1–72.3)^g^	71.5 (68.9–74.1)	70.6 (69.1–72.2)	70.8 (68.9–72.7)
Hearing loss							
Diagnosed	603	9.1 (8.4–9.8)	11 (8–14.9)	9 (8.3–9.7)	10 (8.4–11.9)	8.8 (7.9–9.8)	9 (7.9–10.3)
Suffer	704	10.6 (9.9–11.3)	15.7 (12.1–20.1)	10.3 (9.6–11.1)^g^	11.8 (10–13.8)	10.2 (9.2–11.3)	10.5 (9.3–11.9)
Not applicable	5340	80.3 (79.4–81.3)	73.3 (68.2–77.9)	80.7 (79.7–81.6)^g^	78.2 (75.7–80.5)	81 (79.6–82.3)	80.5 (78.7–82.1)

^a^Up to high school diploma or equivalent.

^b^Certificate or diploma, could be from a registered apprenticeship, or other trade, college, CEGEP or other non-university, university below bachelor's level.

^c^Atlantic provinces include New Brunswick, Nova Scotia, Prince Edward Island and Newfoundland Labrador.

^d^HSD highly sleep disturbed, included responses 8, 9 or 10 on the 11-point numeric scale where 0 was equivalent to not at all sleep disturbed and 10 was equivalent to extremely sleep disturbed.

^e^Highly sensitive to noise, included responses 8, 9 or 10 on the 11-point numeric scale where 0 was equivalent to not at all noise sensitive and 10 was equivalent to extremely sensitive to noise.

^f^Self-reported as First Nation, Métis, Inuk (Inuit).

^g^Significantly different from Indigenous (p < 0.05).

^h^Significantly different from Rural/remote regions (p < 0.05).

E Coefficient of variation was between 16.6 and 33.3%, interpret with caution due to the high sampling variability.

F Coefficient of variation was greater than 33.3%, data could not be released due to questionable validity.

Prevalence of heart disease (including high blood pressure), anxiety or depression, sleep disorder, and hearing loss were reported in CPENS. Diagnosed heart disease (19%) was similar among both Indigenous and non-Indigenous groups and across geographic regions. Diagnosed anxiety or depression was reported by 20.4% overall, with a higher proportion reported among Indigenous people (30.2%) versus non-Indigenous respondents (19.9%), p < 0.05. Similarly, for diagnosed sleep disorders (11.5%) and hearing loss (9.1%), a higher proportion diagnosed with these conditions were reported among Indigenous people (14.6% and 11%, respectively).

**Figure 1:**
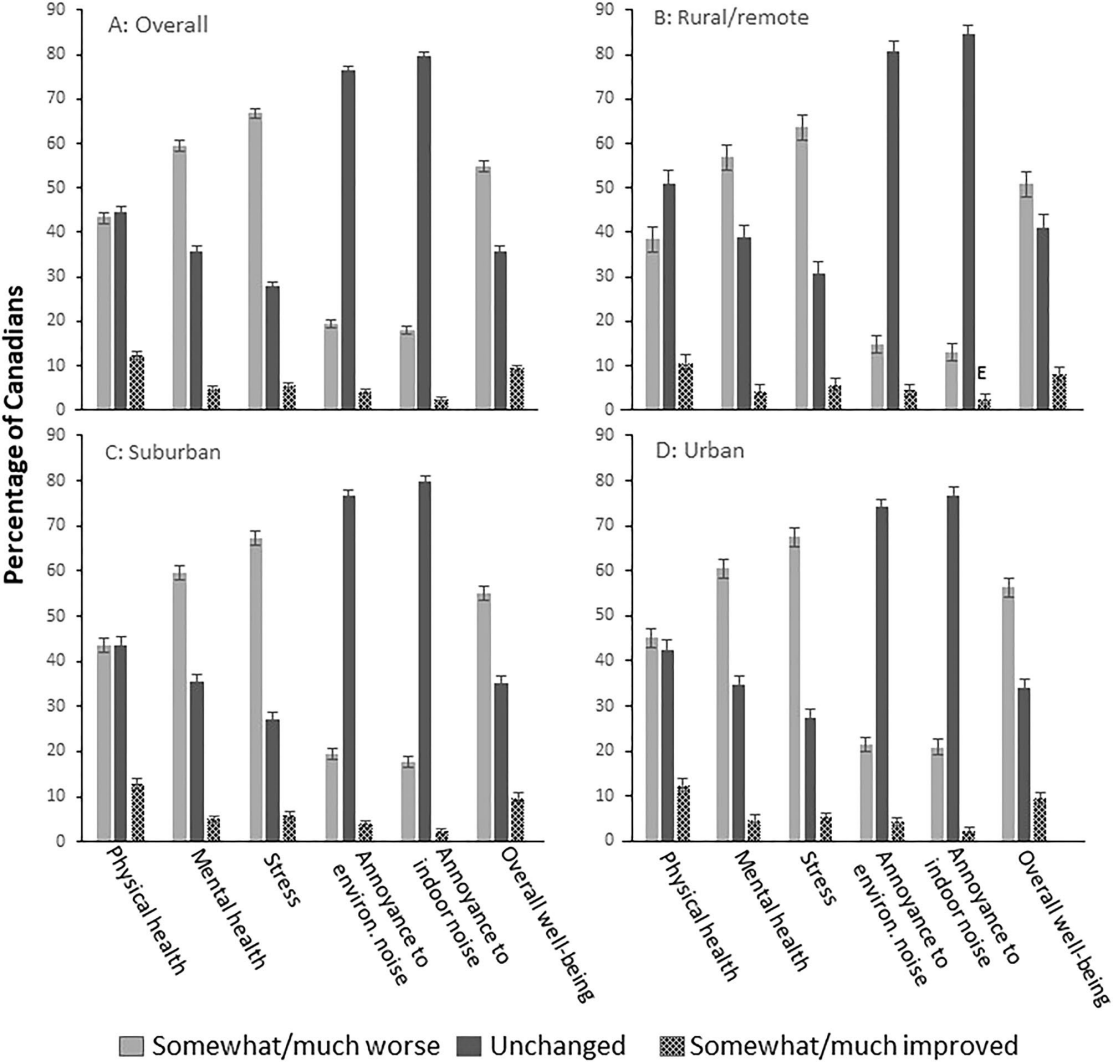
Reported impact of COVID-19 on health-related outcomes and noise annoyance by geographic regions. (**A**) Overall, (**B**) Rural/remote regions, (**C**) Suburban regions, (**D**) Urban regions. Comparisons between geographic regions for each of the health-related outcomes and noise annoyance impacted by COVID-19 was carried out. Significantly higher (p < 0.05) prevalence of “somewhat/much worse” were observed in Suburban and Urban areas compared to Rural/remote areas in Physical health, Overall well-being, Annoyance toward environmental and indoor noise; Significantly lower (p < 0.05) prevalence of “unchanged” were observed in Suburban and Urban areas compared to Rural/remote areas in Physical health, Overall well-being, Annoyance toward environmental and indoor noise. Proportion of “somewhat/much improved” remained statistically similar across geographic regions in each outcome variable impacted by COVID-19.

Figure 1 presents the prevalence of participants who reported “somewhat/much worse”, “unchanged” and “somewhat/much improved” in each of the health related outcomes and noise annoyance variables impacted by the pandemic as a function of geographic region. Overall, as well as in each geographic region, a higher proportion of participants reported that the pandemic had worsened their mental health, stress in their life and overall well-being. Annoyance toward indoor noise and environmental noise were unchanged for the majority of people, regardless of geographic region.

**Figure 2:**
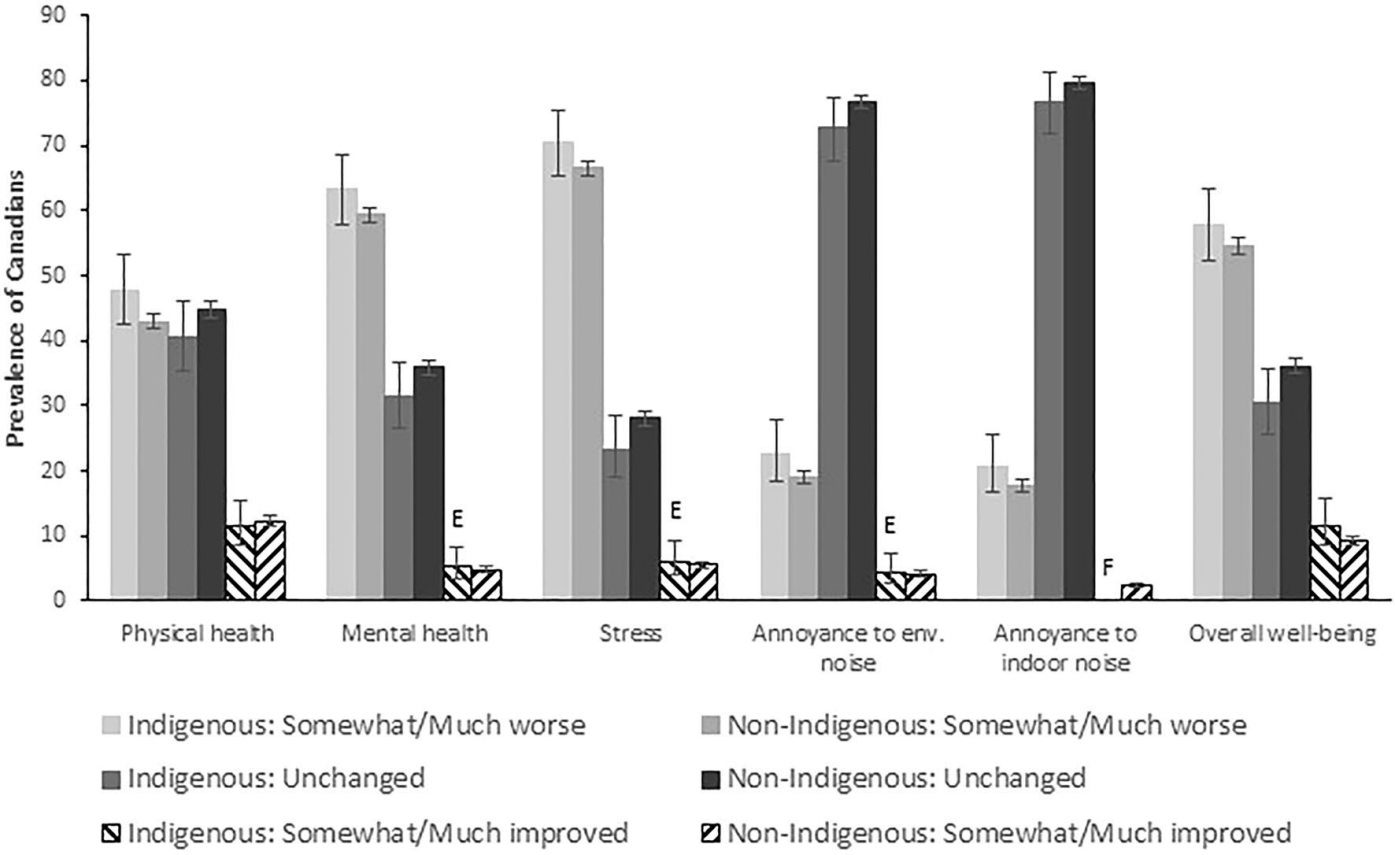
Reported impact of COVID-19 on health-related outcomes and noise annoyance by Indigenous status. Comparisons between Indigenous and non-Indigenous groups for each health-related outcome and noise annoyance measure impacted by COVID-19 by classification was carried out. Prevalence of “somewhat/much worse” in each outcome variable impacted by COVID-19 was statistically similar between Indigenous and non-Indigenous groups. Similar findings for the “unchanged” and “somewhat/much improved” classifications in each outcome variable. E coefficient of variation was between 16.6 and 33.3%, interpret with caution due to the high sampling variability; F coefficient of variation was greater than 33.3%, data could not be released due to questionable validity.

Figure 2 presents the same results as Fig. 1, but is based on Indigenous status. In each of the health related outcomes and noise annoyance variables impacted by COVID-19, Indigenous respondents reported a higher proportion of feeling somewhat/much worse than their non-Indigenous counterparts, though these differences were not statistically significant.

### Multivariate logistic regression for all COVID-19 outcomes

**Figure 3:**
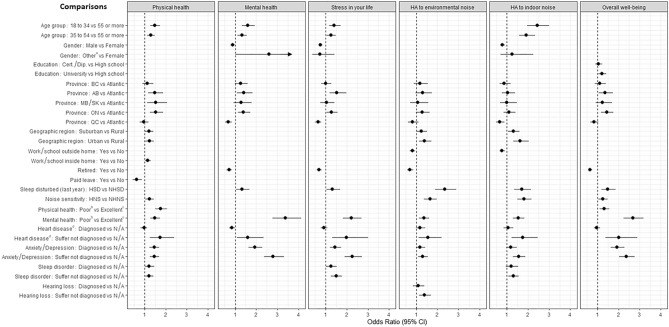
Multivariate logistic regression model as determined by stepwise regression. For each COVID-19 outcome the probability of “Somewhat/much worse” is modelled against “unchanged, somewhat/much improved”. Odds ratio (95% confidence interval CI) is always compared to the reference category as specified in the comparison list. Odds ratios (95% CI) presented are for the variables that remained in the final multivariate logistic regression model. Income, Indigenous status, Unemployed or Other work status did not enter any of the final multivariate logistic regression models. Confidence intervals that do not include 1 indicate a statistical significance of p < 0.05. For the outcome “Mental health”, when comparing genders “Other vs Females” the upper 95% confidence limit was equal to 6.73. It was suppressed here in order to better read the confidence intervals of the other odds ratios. ^a^Includes “Other and prefer not to say”; ^b^Includes “Poor and fair”; ^c^Includes “Excellent, Good, and Very good”; ^d^Including high blood pressure. *AB* Alberta, *BC* British Columbia, *HA* highly annoyed, *HBP* high blood pressure, *HNS* highly noise sensitive, *HSD* highly sleep disturbed, *NHNS* not highly noise sensitive, *NHSD* not highly sleep disturbed, *MB* Manitoba, *ON* Ontario, *QC* Quebec, *SK* Saskatchewan. HSD and HNS included responses 8, 9 or 10 on the 11-point numeric scale where 0 was equivalent to not at all sleep disturbed/noise sensitive and 10 was equivalent to extremely sleep disturbed/sensitive to noise. The NHSD or NHNS included responses 0–7. Atlantic provinces include New Brunswick, Newfoundland and Labrador, Nova Scotia, and Prince Edward Island.

The univariate results for all six outcomes evaluated are presented in Table S1. All variables were included in the stepwise regression method. A stepwise multivariate logistic regression model was used to model the prevalence of reporting “somewhat/much worse” with respect to each of the health-related outcomes and noise annoyance variables impacted by the pandemic. As shown in Fig. 3, certain variables entered all models, such as province, self-reporting one’s overall mental health as fair/poor, heart disease (including high blood pressure) and being diagnosed with, or suffering from anxiety/depression. In each of these variables a similar interpretation of outcomes was observed as in the univariate analysis. In particular, Alberta and Ontario consistently reported higher odds of feeling “somewhat/much worse” with respect to physical and mental health, stress in their life and overall well-being compared to the Atlantic provinces; those living in Quebec had similar (or lower odds) as the Atlantic provinces in all health-related outcomes and noise annoyance variables impacted by the pandemic. Individuals who indicated that their general mental health was fair/poor had significantly higher odds of feeling “somewhat/much worse” in all six evaluated outcomes. The same patterns existed among participants who self-identified as suffering from heart disease. Finally, those reporting to be diagnosed or suffering from anxiety/depression (without a diagnosis) also had higher odds of reporting that their physical and mental health were “somewhat/much worse” due to the pandemic.

Other notable observations included the influence of age, where younger respondents (18 to 34, and 35 to 54) had higher odds of reporting “somewhat/much worse” with respect to physical and mental health, annoyance toward indoor noise and stress in their life. Gender, education, geographic region and the location of work/school influenced the odds on some, but not all of the evaluated outcomes.

High sleep disturbance in general over the previous 12 months at home was associated with significantly higher odds of reporting “somewhat/much worse” on all measures other than physical health. Significantly higher odds of reporting “somewhat/much worse” physical health, annoyance toward environmental and indoor noise and overall well-being was observed among respondents who were categorized as highly sensitive to noise. Individuals who rated their physical health as fair/poor for someone their age had higher odds of reporting that the pandemic made their physical health and overall well-being “somewhat/much worse”. Finally, individuals who rated their mental health as fair/poor had higher odds of reporting “somewhat/much worse” on all measures due to the pandemic.

## Discussion

Depending on the outcome evaluated, between 43 and 67% of respondents reported that their physical health, mental health, stress and overall well-being worsened due to the pandemic. Of these measures, stress was most affected, followed by mental health and then overall well-being. Worsened mental health and stress was most evident among young adults below the age of 35 years. Although males were less impacted than females on both measures, reporting worsened mental health was highest among Canadians who preferred not to indicate their gender, or indicated their gender as other than male or female. These findings may tentatively support the suggestion that some minority groups might be more susceptible to pandemic affects on mental health^17^. Being retired may have served some level of protection from pandemic-related impacts on mental health, stress and overall well-being insofar as the odds of reporting these outcomes as worse due to the pandemic were lower among retired respondents. Under some circumstances, retirement may have shielded Canadians from the burden of uncertainty around economic pressure from job insecurity. It is also not surprising that the pandemic’s toll on mental health, stress, and overall well-being was more pronounced among Canadians who reported to be highly sleep disturbed (in general) over the previous 12 months and among those who reported to have fair/poor mental health.

Our findings are generally consistent with results from Statistics Canada’s Canadian Perspectives Survey Series (CPSS) where COVID-19-related adverse impacts on reported mental health were more pronounced among younger adults when compared to pre-pandemic surveys^18^. The observation that the pandemic had disproportionate impacts on young adults was also made by the British Columbia Centres for Disease Control^17^. Data from both CPENS and CPSS showed noteworthy affects of the pandemic on rated physical health, where the current survey indicated that respondents were as likely to indicate worsening physical health as they were to indicate that it had not changed. In the Statistics Canada’s survey, the prevalence of rating physical health as very good or excellent increased by nearly 10% during the pandemic compared to pre-pandemic levels^18^. This is similar to the 12% who reported improved physical health in the current survey. One might speculate that stay-at-home policies provided greater opportunities for exercise. Indeed, it has been reported that the demand for exercise equipment during the pandemic increased substantially^24^. This would, however, conflict with the finding that there was a slight increase in the odds of reporting worsened physical health among respondents who indicated that they worked, or did their schooling from home. Changes in diet and alcohol consumption may be factors that could have influenced physical health in some individuals^25, 26^.

For its association with other health conditions and the World Health Organization’s characterisation of annoyance as a health effect of environmental noise, Health Canada views changes in noise annoyance as a *potential* risk factor of adverse health^27^. The vast majority of respondents surveyed in CPENS reported that the pandemic did not affect their annoyance toward environmental and indoor noise. However, approximately 1 in 5 Canadians described their annoyance as somewhat or much worse. The modelling results showed that the impacts of the pandemic on annoyance were more pronounced among Canadians living in suburban and urban areas, below the age of 55 years, not working or schooling from home, reporting to be highly sleep disturbed, highly noise sensitive, suffering from anxiety and/or depression, or reporting to have fair or poor mental health. These observations may, at least partially, account for the apparent increase in annoyance in CPENS, when compared to previous national surveys conducted in Canada^28^. Future surveillance would be required to determine if the increase in CPENS is transient or long-lasting.

As anticipated, and consistent with other observations^17, 18, 29–31^ the pandemic was reported to have a much stronger effect on other measures of health, stress and overall well-being. With respect to the other evaluated measures of health, it was hypothesized that Indigenous respondents may be more likely to report that the pandemic worsened mental health, physical health, stress and overall well-being because of factors including, but not limited to a kinship-oriented culture that embraces physical togetherness. Some public health measures, which at times included an emphasis on physical distancing, and/or restrictions on household gatherings, may have adversely impacted traditional Indigenous practices/ceremonies. Furthermore, stress associated with simultaneously caring for, and protecting Elders and Knowledge Keepers from, exposure to COVID-19 have been identified as issues affecting Indigenous Peoples during the pandemic^21^. In the current survey, Indigenous status was unrelated to any of the measures considered in both the unadjusted and adjusted models. However, it should be underscored that CPENS did not permit a comprehensive evaluation of the potential effects of COVID-19 on Indigenous Canadians. The three Territories were not included in the sampling design due to very low overall population density. Furthermore, the survey was available in English and French only and this may have excluded participation from some Indigenous Canadians that communicate in another language. CPENS did not evaluate variation in access to health care, remote learning/employment opportunities, and one’s ability to follow public health measures (e.g. frequent hand washing), all of which have since been identified in relation to the pandemic by Indigenous Peoples of Canada^21^. Future research in this area should be conducted in close partnership with Indigenous leaders across Canada, with questionnaire content developed and administered by Indigenous Peoples of Canada. Finally, despite oversampling Indigenous Canadians, our sample did not permit a separate analysis of First Nation, Métis and Inuk (Inuit) respondents. These shortcomings could be addressed in future surveys.

A novel contribution of CPENS is that it permitted an analysis of self-reported health by geographic region. In the unadjusted models, the data consistently indicated that Canadians living in rural/remote areas had slightly *lower* odds of reporting that COVID-19 worsened the six measures evaluated. In the fully adjusted models, the differences observed in rural/remote areas were only retained for physical health and the two measures of annoyance. The odds of reporting that the pandemic worsened these three measures were significantly reduced in rural/remote areas. These results would suggest that living in rural areas may have provided a buffer against a worsening of health due to the pandemic. Although it is challenging to compare geographic regional differences across countries, these observations appear to be at odds with reports that depression and/or anxiety were lower among urban (in comparison to rural) inhabitants during the pandemic among medical health workers^32^ and college students in China^33^.

CPENS has the strength of providing an overview of how the pandemic was perceived to influence self-reported health among a large representative sample of the general population. The survey also benefits from considering a number of variables that may impact health. Nevertheless, the results are limited insofar as it was outside the scope of CPENS to evaluate health with validated psychometric tools. The assessment of the pandemic’s impact on health was also incomplete inasmuch as CPENS did not include content related to the pandemic’s effect on one’s relationships, pressures related to caring for children/family, employment loss, and if one was directly, or had family/friends, diagnosed with and/or required treatment for COVID-19 symptoms. The current survey did not include Canadians living in long-term care facilities, who experienced significant challenges directly related to COVID-19^34^, or individuals under the age of 18 years, which is an age group with reported deterioration in mental health and stress from COVID-19^19^. It should also be underscored that cross-sectional designs, like CPENS, where data is collected at a single point in time, do not permit statements of causality between the variables evaluated and their potential influence on health. Moreover, it is acknowledged that their can exist a bidirectional relationship between some of the evaluated outcomes and the individual variables included in the models. For instance, stress in one’s life could certainly influence sleep and vice versa. Likewise, it can be difficult to disentangle one’s reported general mental and physical health (overall) and how the pandemic may have exacerbated these same measures. These uncertainties are not unique to CPENS, but apply to cross-sectional designs in general. Despite the noted limitations, CPENS provided an overall assessment of the pandemic’s effect on health and identifies several variables that could be explored further. The results provide some insights that can inform more thorough surveillance in the future, which will continue for some time as scientists attempt to document the effects of the pandemic on the health of Canadians.

In conclusion, the analysis of the data collected in CPENS suggests a high prevalence of Canadians’ self-reported mental health, stress and overall well-being worsened as a result of the pandemic. There was less of an impact on physical health and an even lower impact on noise annoyance. There was no evidence in the data to suggest disproportionate affects on Indigenous Canadians, but there was some indication that Canadians living in rural/remote areas were less likely to report worsened health status, when compared to other geographic areas. This research reflects, and is limited to, responses from Canadians during the third wave of the pandemic in Canada. How the results may have changed throughout the course of the pandemic is unknown, especially as some provinces have recently declared the onset of a seventh wave. Future research in this area could provide the data to determine if the observations on the reported measures of health are temporary, or long-lasting and if widespread vaccine distribution had observable benefits on the evaluated outcomes. Finally, given the noted provincial differences observed in the current study, valuable insights may be gleaned by examining how variation in the stringency of the public health measures applied throughout Canada may have influenced changes in self-reported health.

## Supplementary Information

Supplementary Information.

## Data Availability

The data file is available through Library and Archives Canada website https://epe.lac-bac.gc.ca/100/200/301/pwgsc-tpsgc/por-ef/health/2021/133-20-e/POR133-20-datatables-EN.csv The survey was originally entitled and administered as the “*Survey of Attitudes Towards Community Noise in Canada*”, changed to the *Canadian Perspectives on Environmental Noise Survey* in publications as the revised title more accurately captures the scope of the survey.
