# Supplementary material for: Implications of the COVID-19 pandemic on self-reported health status and noise annoyance in rural and non-rural Canada
Source: Sci Rep. 2022 Sep 24;12:15945. doi: 10.1038/s41598-022-19907-w (PMC9509391; doi:10.1038/s41598-022-19907-w)
Supplement: Supplementary file 1 — Supplementary Information. [file 41598_2022_19907_MOESM1_ESM.docx]

**Univariate logistic regression models**

The univariate results for all six outcomes evaluated are presented in Supplemental Table s1. It is important to keep in mind that the odds ratios based on the univariate logistic regression model are not adjusted for other sources of variability. Some of the notable observations are related to age, where the two younger age groups (18 to 34 and 35 to 54 years old) consistently had higher odds of feeling “somewhat/much worse” with respect to all health-related outcomes and noise annoyance variables impacted by the COVID-19 pandemic. Also, participants who identified their gender as “other/prefer not to say” had a significantly higher prevalence of feeling “somewhat/much worse” with respect to physical health, mental health and annoyance toward indoor noise compared to both females and males. Females reported significantly higher odds of feeling “somewhat/much worse” compared to males with respect to mental health, annoyance toward indoor noise and stress in their life. Individuals with up to high school diploma or equivalent reported higher odds of feeling “somewhat/much worse” with respect to physical health compared to those with a university certificate, diploma or Bachelor’s degree (or higher). In addition, those who reported having a certificate/diploma below bachelor's level reported lower odds of annoyance toward indoor noise compared to respondents with a Bachelor’s degree or higher (data not shown). Individuals from lower income homes (less than $40K per year) reported higher odds of feeling “somewhat/much worse” with respect to physical health and annoyance toward indoor noise compared to the highest household income group with $150K or more per year. In addition, participants who reported a household income between $40K and <$80K reported lower odds of feeling “somewhat/much worse” with respect to mental health, annoyance toward environmental noise and indoor noise, stress in their life and overall well-being compared to the reference group households with less than $40K per year. Participants with household incomes between $80K and <$150K per year also had significantly lower odds of feeling “somewhat/much worse” with respect to mental health, annoyance toward indoor noise and overall well-being compared to the reference group households with less than $40K per year. No significant differences were observed in the proportion of Indigenous and non-Indigenous participants with respect to all health-related outcomes and noise annoyance variables affected by the COVID-19 pandemic.

When considering the participant’s province, those living in Alberta and Ontario consistently reported higher prevalence rates of feeling “somewhat/much worse” with respect to physical and mental health, annoyance toward environmental and indoor noise, stress in their life and overall well-being. Respondents living in Quebec and the four Atlantic Provinces consistently reported lower prevalence rates in all health-related outcomes and noise annoyance variables affected by the COVID-19 pandemic. Similarly, urban and suburban areas constantly had higher odds of feeling “somewhat/much worse” compared to those living in rural/remote areas with respect to physical health, mental health, annoyance toward indoor and environmental noise, stress in their life and overall well-being.

Working or attending school outside the home was associated with higher odds of feeling “somewhat/much worse” with respect to mental health, stress in ones life and overall well-being. Reporting to work or attending school inside the home or being unemployed was significantly associated with increased odds of feeling “somewhat/much worse” in all six health-related outcomes and noise annoyance variables impacted by the COVID-19 pandemic. Retirement was associated with lower odds of feeling “somewhat/much worse” in all six health-related outcomes and noise annoyance variables affected by COVID-19 pandemic. Those who reported being on paid leave (e.g., sick leave, maternity, disability) were associated with higher odds of feeling “somewhat/much worse” with respect to mental health, annoyance toward indoor noise, stress in their life, and overall well-being.

Participants who self-identified as being highly sleep disturbed (in general) over the previous 12 months, or highly sensitive to noise had significantly higher odds of reporting feeling “somewhat/much worse” with respect to all health-related outcomes and noise annoyance variables, compared to those who did not self-identify as being highly sleep disturbed or highly sensitive to noise. Similarly, individuals who rated their overall physical and mental health as fair/poor had significantly higher odds of reporting feeling “somewhat/much worse” with respect to all health-related outcomes and noise annoyance variables impacted by the COVID-19 pandemic.

The four health conditions considered in this survey (heart disease including high blood pressure, anxiety/depression, sleep disorder, hearing loss) were classified as diagnosed, suffer from but not diagnosed, and does not apply. The “does not apply” category was taken as the reference category. Those who self-identified as diagnosed or suffering from anxiety/depression or sleep disorder were significantly more likely to report feeling “somewhat/much worse” with respect to all health-related outcomes and noise annoyance variables impacted by the COVID-19 pandemic. With respect to heart disease, only those who identified as suffering from this condition (but not diagnosed) consistently reported significantly higher odds of feeling “somewhat/much worse” with respect to all health-related outcomes and noise annoyance variables impacted by COVID-19 pandemic. Finally, those who reported they suffer from (but are not diagnosed with) hearing loss had significantly higher odds of feeling “somewhat/much worse” with respect to annoyance toward environmental noise and overall well-being compared to those who did not report suffering from hearing loss.

Supplemental table s1. Unadjusted odds ratios for reporting evaluated outcomes as somewhat or much worse due to the COVID-19 pandemic

|  |  | Self-reported outcome | | | | | |
| --- | --- | --- | --- | --- | --- | --- | --- |
| Variable^a^ | Categories | Physical health | Mental health | Annoyance toward environmental noise | Annoyance toward indoor noise | Stress in your life | Overall well-being |
| Age group (years) | 18 to 34 | 1.86 (1.64 - 2.1)^g^ | 2.88 (2.53 - 3.28)^g^ | 1.26 (1.08 - 1.46) ^g^ | 2.62 (2.23 - 3.08) ^g^ | 2.21 (1.93 - 2.52) ^g^ | 2.01 (1.77 - 2.27) ^g^ |
|  | 35 to 54 | 1.45 (1.29 - 1.63) ^g^ | 1.98 (1.76 - 2.22)^g^ | 1.18 (1.02 - 1.37) ^f^ | 1.87 (1.59 - 2.2) ^g^ | 1.83 (1.62 - 2.06) ^g^ | 1.64 (1.46 - 1.83) ^g^ |
|  | 55+ | Reference | Reference | Reference | Reference | Reference | Reference |
| Gender | Female | Reference | Reference | Reference | Reference | Reference | Reference |
|  | Male | 1.05 (0.95 - 1.16) | 0.87 (0.78 - 0.96) ^g^ | 0.92 (0.81 - 1.04) | 0.71 (0.62 - 0.8) ^g^ | 0.75 (0.68 - 0.83) ^g^ | 0.92 (0.84 - 1.02) |
|  | Other/pref not say | 1.62 (1.06 - 2.47) ^f^ | 2.69 (1.58 - 4.58) ^g^ | 0.94 (0.55 - 1.61) | 1.72 (1.09 - 2.73) ^f^ | 0.92 (0.58 - 1.44) | 1.18 (0.77 - 1.82) |
| Education | High school/eqival | Reference | Reference | Reference | Reference | Reference | Reference |
|  | Cert/Dip <bachelor | 0.82 (0.71 - 0.94) ^g^ | 0.83 (0.72 - 0.96) ^f^ | 1.09 (0.91 - 1.3) | 0.85 (0.71 - 1.02) | 0.97 (0.83 - 1.12) | 0.88 (0.77 - 1.01) |
|  | Bachelor/post grad | 0.8 (0.7 - 0.91) ^g^ | 0.89 (0.78 - 1.03) | 1.05 (0.89 - 1.25) | 1.02 (0.86 - 1.21) | 0.98 (0.85 - 1.13) | 0.95 (0.83 - 1.08) |
| Household  Income | < $40K | Reference | Reference | Reference | Reference | Reference | Reference |
|  | $40K - <$80K | 0.89 (0.76 - 1.04) | 0.79 (0.68 - 0.93) ^g^ | 0.82 (0.68 - 1) ^f^ | 0.68 (0.56 - 0.83) ^g^ | 0.84 (0.71 - 0.99) ^f^ | 0.77 (0.66 - 0.9) ^g^ |
|  | $80K - <$150K | 0.91 (0.79 - 1.06) | 0.85 (0.73 - 0.99) ^f^ | 0.9 (0.75 - 1.08) | 0.73 (0.61 - 0.88) ^g^ | 0.93 (0.79 - 1.09) | 0.85 (0.73 - 0.99) ^f^ |
|  | > $150K | 0.8 (0.67 - 0.95) ^f^ | 0.88 (0.74 - 1.05) | 0.86 (0.69 - 1.06) | 0.75 (0.61 - 0.93) ^g^ | 1.03 (0.85 - 1.24) | 0.91 (0.76 - 1.08) |
| Indigenous status | Indigenous^b^ | 1.21 (0.97 - 1.52) | 1.19 (0.94 - 1.5) | 1.25 (0.95 - 1.63) | 1.21 (0.91 - 1.6) | 1.21 (0.94 - 1.55) | 1.14 (0.91 - 1.44) |
|  | Non-Indigenous | Reference | Reference | Reference | Reference | Reference | Reference |
| Province | BC | 1.09 (0.89 - 1.34) | 1.18 (0.96 - 1.45) | 1.42 (1.09 - 1.86) ^g^ | 0.98 (0.75 - 1.29) | 1.04 (0.84 - 1.28) | 1.1 (0.9 - 1.35) |
|  | AB | 1.5 (1.22 - 1.86) ^g^ | 1.47 (1.19 - 1.82) ^g^ | 1.5 (1.14 - 1.98) ^g^ | 1.17 (0.89 - 1.54) | 1.62 (1.29 - 2.04) ^g^ | 1.46 (1.18 - 1.8) ^g^ |
|  | MB/SK | 1.31 (1 - 1.72) | 1.13 (0.86 - 1.48) | 1.23 (0.86 - 1.75) | 1.01 (0.71 - 1.44) | 1.03 (0.78 - 1.35) | 1.15 (0.88 - 1.5) |
|  | ON | 1.53 (1.28 - 1.83) ^g^ | 1.4 (1.17 - 1.67) ^g^ | 1.48 (1.17 - 1.87) ^g^ | 1.25 (1 - 1.58) | 1.36 (1.13 - 1.63) ^g^ | 1.46 (1.22 - 1.73) ^g^ |
|  | QC | 0.9 (0.74 - 1.1) | 0.69 (0.57 - 0.83) ^g^ | 1 (0.76 - 1.3) | 0.81 (0.62 - 1.06) | 0.68 (0.55 - 0.82) ^g^ | 0.83 (0.68 - 1) ^f^ |
|  | NB/NS/PE/NL | Reference | Reference | Reference | Reference | Reference | Reference |
| Geographic area | Rural/remote | Reference | Reference | Reference | Reference | Reference | Reference |
|  | Suburban | 1.24 (1.08 - 1.42)^g^ | 1.12 (0.98 - 1.28) | 1.41 (1.17 - 1.69) ^g^ | 1.47 (1.21 - 1.78) ^g^ | 1.16 (1.01 - 1.34) ^f^ | 1.19 (1.04 - 1.36) ^f^ |
|  | Urban | 1.32 (1.14 - 1.53) ^g^ | 1.16 (1 - 1.34) ^f^ | 1.59 (1.31 - 1.93) ^g^ | 1.79 (1.46 - 2.19) ^g^ | 1.18 (1.01 - 1.37) ^f^ | 1.25 (1.08 - 1.44) ^g^ |
| Work/attend school outside home | Yes | 0.97 (0.88 - 1.07) | 1.26 (1.14 - 1.4) ^g^ | 0.87 (0.77 - 0.99) ^f^ | 0.87 (0.77 - 1) ^f^ | 1.24 (1.11 - 1.38) ^g^ | 1.16 (1.05 - 1.28) ^g^ |
|  | No | Reference | Reference | Reference | Reference | Reference | Reference |
| Work/attend school inside home | Yes | 1.34 (1.21 - 1.48) ^g^ | 1.32 (1.19 - 1.46) ^g^ | 1.22 (1.07 - 1.38) ^g^ | 1.55 (1.37 - 1.76) ^g^ | 1.31 (1.18 - 1.46) ^g^ | 1.23 (1.11 - 1.36) ^g^ |
|  | No | Reference | Reference | Reference | Reference | Reference | Reference |
| Retired | Yes | 0.61 (0.54 - 0.69) ^g^ | 0.42 (0.37 - 0.47) ^g^ | 0.71 (0.61 - 0.83) ^g^ | 0.49 (0.41 - 0.59) ^g^ | 0.46 (0.41 - 0.52) ^g^ | 0.51 (0.45 - 0.57) ^g^ |
|  | No | Reference | Reference | Reference | Reference | Reference | Reference |
| Unemployed | Yes | 1.48 (1.24 - 1.77) ^g^ | 1.7 (1.4 - 2.06) ^g^ | 1.58 (1.29 - 1.93) ^g^ | 1.71 (1.4 - 2.1) ^g^ | 1.64 (1.33 - 2.01) ^g^ | 1.56 (1.3 - 1.88) ^g^ |
|  | No | Reference | Reference | Reference | Reference | Reference | Reference |
| Other | Yes | 1.11 (0.85 - 1.45) | 1.01 (0.77 - 1.33) | 1.01 (0.72 - 1.42) | 0.82 (0.56 - 1.18) | 1 (0.75 - 1.34) | 0.93 (0.71 - 1.22) |
|  | No | Reference |  |  |  |  |  |
| On paid leave (sick leave, maternity, disability) | Yes | 1.03 (0.77 - 1.39) | 2.13 (1.51 - 3) ^g^ | 1.22 (0.85 - 1.74) | 1.6 (1.14 - 2.25) ^g^ | 1.57 (1.11 - 2.22) ^g^ | 1.79 (1.31 - 2.46) ^g^ |
|  | No | Reference | Reference | Reference | Reference | Reference | Reference |
| Sleep disturbed | Highly sleep disturbed^c^ | 1.55 (1.29 - 1.85) ^g^ | 1.54 (1.27 - 1.87) ^g^ | 2.58 (2.13 - 3.12) ^g^ | 2.35 (1.93 - 2.86) ^g^ | 1.62 (1.31 - 1.99) ^g^ | 1.82 (1.5 - 2.2) ^g^ |
|  | Not highly sleep disturbed^d^ | Reference | Reference | Reference | Reference | Reference | Reference |
| Noise Sensitivity | Highly sensitive to noise^c^ | 1.28 (1.11 - 1.48) ^g^ | 1.27 (1.1 - 1.47) ^g^ | 1.84 (1.56 - 2.16) ^g^ | 2.08 (1.77 - 2.45) ^g^ | 1.29 (1.11 - 1.51) ^g^ | 1.4 (1.21 - 1.62) ^g^ |
|  | Not highly sensitive to noise^d^ | Reference | Reference | Reference | Reference | Reference | Reference |
| General physical health for one’s age | Fair/poor | 2.29 (2 - 2.61) ^g^ | 1.75 (1.52 - 2.01) ^g^ | 1.23 (1.05 - 1.44) ^f^ | 1.57 (1.34 - 1.84) ^g^ | 1.86 (1.59 - 2.16) ^g^ | 2.07 (1.8 - 2.37) ^g^ |
|  | Excellent/very good/good | Reference | Reference | Reference | Reference | Reference | Reference |
| General mental health | Fair/poor | 2.52 (2.23 - 2.85) ^g^ | 6.77 (5.71 - 8.02) ^g^ | 1.84 (1.6 - 2.11) ^g^ | 2.56 (2.23 - 2.94) ^g^ | 4.07 (3.46 - 4.79) ^g^ | 4.66 (4.03 - 5.38) ^g^ |
|  | Excellent/very good/good | Reference | Reference | Reference | Reference | Reference | Reference |
| Heart disease including high blood pressure | Diagnosed | 0.83 (0.74 - 0.95) ^g^ | 0.61 (0.54 - 0.69) ^g^ | 1.09 (0.93 - 1.27) | 0.73 (0.61 - 0.87) ^g^ | 0.72 (0.64 - 0.82) ^g^ | 0.8 (0.71 - 0.91) ^g^ |
|  | Suffer^e^ | 2.11 (1.59 - 2.79) ^g^ | 1.86 (1.36 - 2.55) ^g^ | 1.92 (1.43 - 2.6) ^g^ | 1.87 (1.38 - 2.53) ^g^ | 2.37 (1.65 - 3.4) ^g^ | 2.74 (1.98 - 3.79) ^g^ |
|  | Does not apply | Reference | Reference | Reference | Reference | Reference | Reference |
| Anxiety or depression | Diagnosed | 2.15 (1.9 - 2.44) ^g^ | 3.48 (3.03 - 3.99) ^g^ | 1.74 (1.5 - 2.03) ^g^ | 2.27 (1.94 - 2.65) ^g^ | 2.69 (2.33 - 3.1) ^g^ | 3.15 (2.76 - 3.59) ^g^ |
|  | Suffer^e^ | 2.13 (1.88 - 2.42) ^g^ | 4.67 (4.03 - 5.42) ^g^ | 1.71 (1.47 - 1.99) ^g^ | 2.4 (2.06 - 2.79) ^g^ | 3.66 (3.14 - 4.28) ^g^ | 3.55 (3.1 - 4.07) ^g^ |
|  | Does not apply | Reference | Reference | Reference | Reference | Reference | Reference |
| Sleep disorder | Diagnosed | 1.52 (1.3 - 1.77) ^g^ | 1.36 (1.16 - 1.59) ^g^ | 1.49 (1.24 - 1.79) ^g^ | 1.42 (1.17 - 1.71) ^g^ | 1.35 (1.14 - 1.59) ^g^ | 1.54 (1.32 - 1.8) ^g^ |
|  | Suffer^e^ | 1.51 (1.33 - 1.72) ^g^ | 1.64 (1.43 - 1.87) ^g^ | 1.47 (1.26 - 1.71) ^g^ | 1.7 (1.45 - 1.98) ^g^ | 1.94 (1.67 - 2.26) ^g^ | 1.71 (1.5 - 1.95) ^g^ |
|  | Does not apply | Reference |  |  |  |  |  |
| Hearing loss | Diagnosed | 1.01 (0.85 - 1.2) | 0.83 (0.7 - 0.98) ^f^ | 0.97 (0.78 - 1.21) | 0.79 (0.63 - 1) | 0.81 (0.68 - 0.97) ^f^ | 0.97 (0.82 - 1.15) |
|  | Suffer^e^ | 1.04 (0.89 - 1.22) | 1.07 (0.91 - 1.25) | 1.4 (1.16 - 1.69) ^g^ | 1.08 (0.88 - 1.32) | 1.11 (0.94 - 1.31) | 1.28 (1.09 - 1.5) ^g^ |
|  | Does not apply | Reference | Reference | Reference | Reference | Reference | Reference |

^a^For each endpoint the probability of “Somewhat/much worse” is modelled against “unchanged, somewhat/much improved”. Odds ratio (95% CI) is always compared to the reference category; ^b^Self-reported as First Nation, Métis, Inuk (Inuit); ^c^Included responses 8, 9 or 10 on the 11-point numeric scale where 0 was equivalent to not at all sleep disturbed/noise sensitive and 10 was equivalent to extremely sleep disturbed/sensitive to noise; ^d^Included responses 0-7 on the 11-point numeric scale where 0 was equivalent to not at all sleep disturbed and 10 was equivalent to extremely sleep disturbed/sensitive to noise; ^e^Respondent indicated to suffer from the condition without having a formal diagnosis from a healthcare practitioner; ^f^p<0.05 when compared to the reference category; ^g^p<0.01 when compared to the reference category. *AB* Alberta, *BC* British Columbia, *MB* Manitoba, *NB* New Brunswick, *NL* Newfound Land & Labrador, *NS* Nova Scotia, *ON* Ontario, *PE* Prince Edward Island, *QC* Quebec, *SK* Saskatchewan
